# Maternal Dietary Intake of Folic Acid, Vitamin B12, and Choline During Pregnancy and Lactation Alters DNA Methylation in Offspring

**DOI:** 10.3390/nu18203306

**Published:** 2026-10-09

**Authors:** Alek J. Abell, Lillian K. Schuler, Nafisa M. Jadavji

**Affiliations:** Division of Molecular and Integrative Physiology, Department of Biomedical Science, School of Medicine, Southern Illinois University Carbondale, Carbondale, IL 62901, USA

**Keywords:** maternal diet, DNA methylation, folic acid, vitamin B12, choline, S-adenosylmethionine, Developmental Origins of Health and Disease

## Abstract

Maternal nutrition during pregnancy and lactation plays an important role in the neurodevelopment of offspring. One-carbon (1C) metabolism includes B-vitamins such as folic acid (vitamin B9) and vitamin B12, and the nutrient choline. Adequate dietary intake of B-vitamins and choline by women prior to getting pregnant and during pregnancy is essential for the neurodevelopment of the infant. The aim of this narrative review is to summarize the current findings on maternal diet intake of B-vitamins and choline and how their intake impacts DNA methylation levels in offspring. From our analysis of clinical and preclinical research, the evidence indicates that maternal intake of 1C nutrients during pregnancy is associated with DNA methylation in offspring during early life; however, it remains unknown whether these changes last past early life. Additionally, the impact on health outcomes requires further investigation. The studies included in our narrative review were heterogeneous and small, and future studies are needed to draw more definitive conclusions. To conclude the review, we discuss opportunities to enhance the scientific rigor of future studies in this field. One point to consider is the inclusion of both female and male offspring in all outcome assessments to facilitate a comprehensive understanding of potential sex-specific effects and to determine how each sex may be differentially impacted by maternal B-vitamin and choline intake during pregnancy and lactation. Additionally, increasing sample size appropriately and initiating multi-center preclinical randomized controlled trials (mpRCTs) could enhance scientific rigor.

## 1. Introduction

One-carbon (1C) metabolism plays an important part in processing and transforming nutrients. Through its two main cycles, the folate and methionine cycles, 1C metabolism uses vitamin B12, choline, and folic acid (vitamin B9), the synthetic form of folate, to perform DNA methylation, nucleotide synthesis, and other key processes in early and later development of the brain [1]. For these crucial processes to be performed during fetal development, there must be sufficient and increased vitamin intake in the mother’s diet to meet the demand of the fetus’s growth. For example, the US recommends that a typical person’s intake of folic acid is around 400 µg per day, while expecting and lactating women are recommended to supplement around 600 µg per day [2]. Choline requirements during pregnancy also increase from 425 to 550 mg per day [3]. Adequate amounts of both choline and folic acid are important to minimize neural tube defects [4,5]. In 1998, the US and Canada implemented mandatory folic acid fortification in order to reduce the number of neural tube defects [6]; other regions in the world have followed suit. Mandates for choline have not been made yet. Many in the field have advocated for implementing stricter guidelines for choline supplementation during pregnancy [7,8]. In addition, genetic polymorphisms of enzymes involved in 1C can also influence neural tube closure [9]. Studies from our group have shown that maternal deficiencies in folic acid and choline can negatively impact health outcomes of offspring later in life after an ischemic stroke [10,11,12,13,14,15].

While 1C metabolism is involved in the replication of DNA with nucleotide synthesis, it also plays an important role in epigenetics and DNA methylation [16,17,18,19,20]. The 1C metabolism pathway of the methionine cycle uses 5-methyltetrahydofolate, that is produced in the folate cycle, and vitamin B12 to convert homocysteine to methionine [21]. Choline can also be used as an alternative methyl donor to convert homocysteine to methionine with the use of betaine and betaine homocysteine methyltransferase (BHMT). Then, S-adenosylmethionine (SAM) donates a methyl group to DNA, therefore performing DNA methylation [18]. SAM specifically attaches the methyl group to a cytosine, resulting in 5-methylcytosine [13,15,19]. The methylated regions of DNA typically inhibit the expression of certain genes, while allowing others to be expressed [22]. Specifically, the methylation of DNA and histones can determine chromosome activation, cell differentiation, and metabolic activity [13,15,19]. Not only can hypomethylation have adverse effects on the development of offspring, but hypermethylation can also impact offspring health and susceptibility to disease [20]. For example, hypermethylation has been linked with Alzheimer’s disease and bipolar disorder, while hypomethylation has been linked with cancer and cardiovascular disease [23,24,25]. Additionally, current research on Alzheimer’s disease has shown that the methylation patterns are sex-dependent, with males and females showing distinct gene methylation and activation [26]. Without sufficient B-vitamin or choline consumption during pregnancy, 1C metabolism cannot function properly, resulting in the inability to produce adequate amounts of SAM to participate in methylation reactions [22]. The exact impact of varying B-vitamin or choline intake during pregnancy on offspring health outcomes is an ongoing area of research.

The Developmental Origins of Health and Disease (DOHaD) hypothesis proposes that environmental exposures during critical periods of development, particularly during prenatal and early postnatal life, can have lasting effects on health and disease risk across the lifespan [27,28,29]. The DOHaD hypothesis has recently been demonstrated to impact high-risk neonates, including infants born preterm, growth-restricted, medically fragile, or requiring complex surgical care, who are particularly vulnerable to nutritional inadequacies during early life [30]. Epigenetic mechanisms, especially DNA methylation, are thought to mediate these long-term effects by altering gene expression without changing the underlying DNA sequence. During fetal development, the epigenome undergoes extensive reprogramming and is highly sensitive to maternal environmental factors, including nutrition, stress, metabolic status, and toxicant exposures. Maternal dietary intake of 1C metabolism nutrients such as folic acid, vitamin B12, and choline is of particular interest because these nutrients provide methyl groups necessary for the synthesis of SAM. Consequently, variations in maternal nutritional status may influence offspring DNA methylation patterns, potentially affecting growth, neurodevelopment, metabolism, and susceptibility to chronic diseases later in life. Human and animal studies have demonstrated associations between prenatal exposures and differential DNA methylation in offspring, supporting the concept of developmental programming; however, relatively few studies have directly linked prenatal exposures, epigenetic alterations, and long-term health outcomes within the same population [27,28,29]. Despite ongoing challenges related to tissue specificity, causality, and the persistence of methylation changes over time, DNA methylation remains one of the most extensively studied and biologically plausible mechanisms underlying the DOHaD paradigm.

This narrative review examines the impact of maternal folic acid, vitamin B12, and choline supplementation and deficiency on offspring epigenetic regulation, with a particular focus on DNA methylation and neurodevelopmental outcomes. We first evaluate evidence from clinical studies involving mothers and their offspring, followed by a synthesis of preclinical research that provides mechanistic insights into the biological pathways linking maternal micronutrient status to epigenetic modifications and neurodevelopment. Clinical and preclinical studies included in this narrative review were not limited by publication date. Clinical and preclinical studies were included and were identified using the PubMed and Web of Science databases. The search terms included maternal nutrition, DOHaD, B-vitamins, folic acid, vitamin B12, offspring DNA methylation, models and human. This narrative review also considers important aspects of scientific rigor and reproducibility in the design and interpretation of maternal–offspring research, with the goal of strengthening the translational relevance of findings across clinical and experimental settings.

## 2. Linking Maternal Dietary Intake of 1C Vitamins and Nutrients to Offspring DNA Methylation in Clinical Data

We evaluated clinical studies that assessed DNA methylation outcomes in offspring for whom maternal use of folic acid, vitamin B12, or choline supplements had been documented during pregnancy. To identify relevant evidence, we conducted a comprehensive literature search without imposing any restrictions on publication year. This search yielded three eligible studies that met our inclusion criteria and examined associations between maternal supplementation and DNA methylation patterns in offspring. A summary of each study is provided below and in **Table 1**.

In a clinical population of 463 mother–infant pairs, the association between maternal dietary intake of folic acid and folate and newborn DNA methylation was measured [31]. During the early stages of pregnancy, levels of maternal folate and folic acid, and homocysteine concentrations were measured. Additionally, folic acid supplement use was self-reported, and maternal and infant methylenetetrahydrofolate reductase (MTHFR) genotype was determined. In offspring, DNA methylation of genes that are involved in fetal growth and neurodevelopment was assessed. These genes included insulin-like growth factor-2 (IGF2), H19, nuclear receptor subfamily 3 group c member 1 (NR3C1), dopamine D4 receptor (DRD4), and serotonin transporter (5-HTT). The study determined that maternal folate deficiency was related to lower DNA methylation levels in newborns. Maternal MTHFR C677T genotype was associated with reduced methylation of genes involved in fetal growth and neuronal developmental genes in offspring, which suggests a genetic influence on 1C metabolism and epigenetic regulation. Maternal folate concentrations, once adjusted for maternal educational levels, smoking, child sex, birth weight, and gestational age at birth, were not associated with newborn DNA methylation. Furthermore, homocysteine levels and folic acid supplement use were not consistently connected with DNA methylation outcomes. A strength of this study would be its large sample size and population design, while limitations include the study’s observational design, which precludes causal inference.

Another study examined the connection between maternal intake of methyl-group donors and DNA methylation within infants using the Maternal Nutrition and Offspring’s Epigenome (MANOE) cohort (*n* = 114) [22]. Maternal dietary and supplemental intakes of betaine, folate, choline, and methionine were all measured before and throughout pregnancy. Infant DNA methylation was also measured in buccal epithelial cells at six months of age, during lactation. This study determined that maternal intake of methyl-group donors during the periconceptional period was connected to DNA methylation within genes that are related to growth, metabolism, and appetite regulation, such as IGF2, retinoid x receptor alpha (RXRA), leptin producing gene (LEP), and DNA methyltransferase 1 (DNMT1). The researchers reported that DNA hypermethylation of RXRA was modulated by maternal dietary intake of choline. Maternal folic acid and folate intakes were negatively connected with the methylation of IGF2 and LEP, genes involved in human growth, metabolism, and energy balance. Positive connections were shown between maternal folate and folic acid and RXRA methylation. These results suggest that maternal nutrition throughout the early stages of pregnancy may influence epigenetic programming within offspring in early life, but the long-term impact remains unknown. Strengths of this study include the assessment of multiple dietary methyl donors and gene-specific methylation analysis. This study did not report locus-specific methylation, just associations. Specific information on specific loci methylation would have been proven to be informative. Other limitations include self-reported dietary intake, which can potentially affect the precision of the findings. Furthermore, offspring methylation was only measured at 6 months of age, and no long-term measurements were made.

A longitudinal study of 24 mother–infant dyads measured DNA methylation at 993 CpG sites using maternal and cord blood [32]. Additionally, maternal 1C metabolites were measured throughout early pregnancy and at the time of delivery. This study determined that several one-carbon metabolites, such as choline, S-adenosylhomocysteine (SAH), and red blood cell (RBC) folate, had changed throughout the course of pregnancy, and vitamin B12 and betaine decreased. Maternal and cord blood metabolite concentrations were associated, while a few CpG sites in infant cord blood were related to metabolite levels. This study showed strengths through its design, but its small sample size and small fraction of the epigenome (993 CpG sites) were significant limitations. Keeping the limitations in mind, the results of this study do add to the growing body of literature that suggests a connection between maternal metabolic status and offspring epigenetic patterns; however, more extensive studies are needed to draw more definite conclusions.

## 3. Mechanistic Evidence from Preclinical Studies Demonstrating How Maternal Diet Impacts Offspring DNA Methylations

Preclinical studies show that the interactions between maternal folic acid and vitamin B12 intake regulate offspring outcomes through epigenetic and metabolic mechanisms. One-carbon metabolism mediated these reactions, while both vitamins are crucial for gene regulation and DNA methylation. A summary of each study is provided below and in **Table 2**.

### 3.1. Interactions Between Maternal Folic Acid and Vitamin B12 Intake

Two studies highlighted the overall consequences of imbalance between folic acid and vitamin B12 [1,20]. Maternal vitamin B12 deficiency mixed with excess folic acid resulted in global DNA hypermethylation while also altering the expression of genes critical for brain development within the offspring [20]. From the study’s findings, excess folic acid cannot compensate for vitamin B12 deficiency and can possibly exacerbate epigenetic expression of neurodevelopment markers brain-derived neurotrophic factor (BDNF), nerve growth factor (NGF), and CREB; these markers are essential for neuronal survival and plasticity [1]. BDNF, NGF, and CREB were normalized by folic acid supplementation, which indicates a complex and dose-dependent interaction between these vitamins. These studies were limited due to their lack of reporting offspring sex and sample size.

These findings were supported by another study which showed that maternal dietary imbalance between folic acid and vitamin B12 affects DNA methylation patterns as well as miRNA expression within both maternal and fetal tissues [33]. These changes suggest impaired methylation capacity, since they were related to disruptions in one-carbon metabolism. Within this study, evidence of multigenerational epigenetic effects was provided, showing that maternal nutrient status can potentially have long-term consequences on gene regulation within the offspring.

### 3.2. Maternal Folic Acid Supplementation

Mandatory folic acid fortification has led to an increase in the prevalence of over-supplementation in participating countries. The Centers for Disease Control recommends 400 µg per day of folic acid daily for adults [36]. Over-supplementation is defined as a consumption of folic acid over 1000 µg per day in adults [37].

In pregnant rats, increasing maternal dietary folic acid intake by 2.5× can change DNA methylation and gene expression, as observed across several tissues, including the brain and liver [21]. Global DNA methylation in brain tissue decreased with later gestational folic acid supplementation. The lowest levels were observed when supplementation occurred during pregnancy. Gene expression changes were more noticeable within liver tissue. The genes that were significantly decreased were estrogen receptor-α (ER-α), glucocorticoid receptor (GR), and peroxisome proliferator-activated receptor-α (PPAR-α). The study’s findings show how maternal folic acid intake ultimately affects epigenetic regulation within the offspring. The study was limited by its lack of sex-specific analyses.

Another study, focusing more on the central nervous system, showed that excessive maternal folic acid supplementation (4 mg/kg from 0.4 mg/kg in control die) can trigger alterations in DNA methylation patterns across brain tissue in the offspring [17]. Both CpG and non-CpG methylation sites were specifically affected. Differential methylation was observed in genes involved in neural development, synaptic function, and candidate autism susceptibility pathways. These changes were linked to alterations in gene expression, which indicates that maternal folic acid intake during gestation can ultimately influence epigenetic regulation within the developing brain. These findings are significant as they suggest that gene regulation related to development might be impacted by folic acid intake during gestation. This study’s main strength was its focus on brain tissue, although its conclusions are limited by its use of a single model system and a relatively small sample size.

These findings were further explored by another study that examined the effects that excessive maternal folic acid (20 mg/kg) intake can have on gene regulation throughout the offspring’s brain tissue [34]. This preclinical study observed modifications in gene expression and chromatin accessibility across multiple brain regions. The pathways related to neurogenesis, neuronal axon myelination, and learning and memory were affected. Within the dentate gyrus, excitatory neurons were found to be particularly sensitive to high maternal folic acid intake. These findings suggest that maternal folic acid levels can alter epigenetic regulation at both transcriptional and chromatin levels in the developing brain. Although this study was limited by its small sample size and the lack of extensive validation for the observed molecular studies, the study is strengthened by the advanced molecular techniques used.

### 3.3. Maternal Choline Supplementation

To determine the role of prenatal choline as a modulator of metabolism, prenatal choline supplementation in drinking water (0.25% choline bitartrate) was administered to pregnant female Wistar rats from day two of gestation until the birth of their offspring [35]. During lactation, choline supplementation was removed from drinking water. Offspring were weaned onto a high-fat diet for 12 weeks. In terms of DNA methylation, the investigators measured the expression of genes involved in metabolic health in the liver. They reported that at post-weaning hepatic gene expression of insulin signaling substrates, *Irs2* (insulin receptor substrate 2) was higher in females, and this continued to 12 weeks, whereas *Irs1* (insulin receptor substrate 1) expression was lower in male offspring only at the 12-week timepoint. When DNA methylation was measured at the 12-week timepoint, *Irs2* was lower in choline-supplemented females. Male offspring with choline supplementation had higher *Irs1* methylation. So, there was an inverse relationship between gene expression and DNA methylation. The authors suggest that the complexity of the phenotypes has been reported in Irs1 knockout mice previously. Overall, the study concluded that maternal choline supplementation improved insulin signaling. A major strength of this study is that both male and female offspring were included in the analysis. The long-term impact of prenatal choline supplementation was partly addressed with the 12-week timepoint measurements, but a longer period, such as 6 months or even a year, would be valuable. There are other studies investigating maternal dietary choline supplementation and how it impacts DNA methylation in the context of maternal obesity, but we did not include them in our review as they include another experimental component, maternal fat, which is not within the scope of this narrative review [38,39].

## 4. Discussion

Maternal dietary intake of 1C nutrients, including folic acid, vitamin B12, and choline, is essential for neural tube closure and the subsequent development of the brain and spinal cord in the developing fetus. It is well established that 1C metabolism contributes to epigenetic regulation through the generation of SAM, the primary methyl donor for DNA methylation. The aim of this narrative review was to examine the effects of maternal folic acid, vitamin B12, and choline supplementation, as well as deficiency, on offspring epigenetic regulation, with particular emphasis on DNA methylation and neurodevelopmental outcomes. Collectively, the available evidence indicates an association that maternal intake of 1C nutrients during pregnancy influences epigenetic regulation in offspring during early life. However, current research is largely limited to observations made within the first days, months, or years after birth, highlighting the need for studies examining the long-term effects of maternal 1C nutrition on offspring health and development. Additionally, there were very few clinical and preclinical studies in this area.

A notable limitation of the studies included in this narrative review is that both clinical and preclinical investigations primarily assessed offspring DNA methylation outcomes at relatively acute postnatal timepoints. In most cases, epigenetic measurements were obtained during the neonatal period or within the first months to years of life. Practical considerations, including funding constraints, study duration, participant retention, and the logistical challenges associated with long-term follow-up, have likely contributed to the predominance of these early-life assessments. Consequently, there remains a significant gap in our understanding of the long-term persistence and biological significance of DNA methylation alterations associated with maternal 1C nutrient status during pregnancy. Longitudinal studies extending into adolescence and adulthood, as well as transgenerational studies, would provide valuable insight into whether epigenetic modifications induced by maternal folic acid, vitamin B12, and other 1C nutrients are stable across the lifespan or they are subject to remodeling during postnatal development. Although DNA methylation is often considered a relatively stable epigenetic mark, accumulating evidence suggests that the epigenome remains responsive to environmental exposures throughout life [40]. Therefore, it is currently unclear whether methylation changes established during fetal development persist indefinitely or gradually diminish as offspring are exposed to new nutritional, environmental, and social influences. This question is particularly relevant in the context of neurodevelopment, which is shaped by a complex interplay of genetic, epigenetic, and environmental factors. Postnatal experiences, including nutrition, socioeconomic conditions, educational opportunities, stress exposure, and other environmental influences, can substantially affect neurodevelopmental trajectories [41]. As a result, the contribution of prenatal epigenetic programming to later neurodevelopmental outcomes may be modified by factors encountered after birth. Understanding the relative influence of prenatal versus postnatal determinants of neurodevelopment remains an important area for future investigation.

The developing brain exhibits remarkable plasticity throughout infancy and childhood [42]. This plasticity raises the intriguing possibility that adverse epigenetic effects associated with maternal one-carbon nutrient deficiencies may not be permanent. For example, in a hypothetical experiment, if an offspring is exposed to inadequate folate or vitamin B12 during fetal development but subsequently receives adequate or enhanced nutritional support during critical postnatal periods, it is conceivable that some of the associated DNA methylation alterations could be partially or fully reversed. Such reversibility has important implications for the design of nutritional interventions and public health strategies aimed at mitigating the consequences of prenatal nutrient deficiencies. However, the extent to which postnatal nutritional supplementation can remodel epigenetic marks established during early development remains poorly understood and these experiments do need to be conducted. Future research should therefore prioritize longitudinal studies that track offspring epigenetic profiles and neurodevelopmental outcomes across multiple life stages. Such investigations would help determine the durability of prenatal nutrition-induced epigenetic modifications, identify critical windows of susceptibility and intervention, and clarify whether postnatal nutritional interventions can modify or reverse epigenetic signatures established during fetal development. Ultimately, these studies will be essential for understanding the long-term implications of maternal one-carbon nutrient intake on offspring health and neurodevelopment across the lifespan.

Although the focus of our narrative review is on understanding the effect of maternal diet on offspring DNA methylation, it is plausible that paternal effects may also be important, especially if epigenetic influences are key factors [43]. While this may be a future area for study, that is outside the scope of this narrative review. However, it is an interesting avenue of research that does need further investigation.

Scientific rigor in both clinical and preclinical studies is being considered increasingly important as there is a movement toward demonstrating the effective use of funds to conduct experiments and translate findings to humans [44]. The preclinical studies presented in this narrative review had several strengths, including strictly controlled dietary interventions and a focus on molecular endpoints such as DNA methylation and gene expression. However, some of the preclinical studies were limited by small sample sizes, inconsistent reporting of sex-specific outcomes, and the lack of direct evidence linking molecular changes to functional phenotypes. Furthermore, the inclusion of female and male offspring was not clear in some studies [45]; the inclusion of both sexes, however, is vital to gain a foundation of the basic mechanism to build off.

To improve scientific rigor, several strategies have been proposed. Multi-center preclinical randomized controlled trials (mpRCTs), modeled after phase III clinical trials, may enhance reproducibility and translational value [46]. Standardized reporting frameworks, such as the ARRIVE (Animal Research: Reporting of In Vivo Experiments) guidelines developed by the UK National Centre for the 3Rs (NC3Rs), also promote transparency and methodological consistency [47]. In addition, educational initiatives such as Reproducibility for Everyone (R4E) provide training in best research practices, including data management, electronic lab notebooks, and data sharing [44]. Despite ongoing challenges, the outlook for preclinical and clinical research remains encouraging in the field of maternal nutrition and offspring health outcomes. Increased emphasis on rigorous methodologies, advanced analytical tools, interdisciplinary collaboration, and open science practices is improving reproducibility and accelerating the translation of scientific discoveries into clinical applications. Ultimately, these efforts will strengthen the reliability and impact of biomedical research.

Overall, the evidence from this narrative review indicates a complex relationship between maternal 1C dietary intake during pregnancy and offspring DNA methylation. This review was limited to a few small and heterogenous studies. While existing studies suggest that maternal nutrition may influence epigenetic programming, the durability of these methylation changes and their relevance to subsequent health outcomes remain unclear. Future longitudinal studies are warranted to elucidate the persistence, functional significance, and clinical implications of these epigenetic modifications. Additionally, studies investigating whether DNA methylation changes impact health outcomes need to be conducted. Given the growing interest in DOHaD, this remains an exciting area of research with substantial opportunities for future discovery.

## Figures and Tables

**Table 1:** Summary of main findings from clinical studies.

Citation and PMID	Study Question(s) Being Investigated	Dietary Manipulation	Study Population	What Countries/Regions Are Considered?	Main Findings
Van Mill [31] PMID: 25392189	Does maternal one-carbon metabolism at early pregnancy influence DNA methylation patterns of selected growth- and neurodevelopment-related genes within newborns?	No experimental dietary manipulation was performed.	463 mother–child pairs	Rotterdam, The Netherlands	In this study, it was found that the maternal MTHFR 677TT genotype was connected with lower DNA methylation within newborn cord blood. Although maternal folate deficiency first appeared to be associated with lower levels of methylation, this effect changed once cofounding factors were adjusted. No significant relationships were discovered for maternal homocysteine levels, folic acid supplement use, or even the newborn’s MTHFR genotype.
Pauwels [22] PMID: 28191262	Did maternal intake of methyl-group donors throughout pregnancy and lactation influence gene-specific DNA methylation within 6-month-old infants?	No experimental dietary manipulation was performed.	114 healthy Caucasian mother–infant pairs	Belgium	This study found that before and early in pregnancy, maternal intake of methyl-group donors was associated with altered DNA methylation of metabolic genes within 6-month-old infants. As for lactation, associations were limited.
Knight [32] PMID: 30442960	Does one-carbon metabolite levels as well as genome-wide DNA methylation change throughout pregnancy, and are maternal and cord blood metabolite concentrations specifically linked to DNA methylation patterns?	Standardized prenatal supplements and 400–800 µg of folic acid were provided daily.	24 pregnant women and their infants	United States	For this study, the main findings showed that five one-carbon metabolites and 993 CpG sites significantly changed throughout pregnancy.

**Table 2:** Summary of main findings from preclinical studies.

Citation and PMID	Study Question(s) Being Investigated	Dietary Manipulation	Timepoint of Dietary Manipulation	Main Findings
Sable [1] PMID: 24257323	In what ways does maternal folic acid and vitamin B12 imbalance throughout pregnancy affect global DNA methylation in the offspring brain and do omega-3 fatty acids normalize these effects?	Normal or excess folic acid, with sufficient or deficient vitamin B12. Some B12-deficient groups received omega-3 fatty acid supplementation. Half of the dams were switched to a control diet after delivery.	Pregnancy	An imbalance in maternal folic acid and vitamin B12 altered global DNA methylation in the offspring brain. The offspring showed reduced methylation at birth and increased methylation in the adult cortex. Prenatal omega-3 fatty acid supplementation partially helped normalize these methylation changes.
Sable [20] PMID: 24462543	In the adult offspring brain, does maternal folic acid and vitamin B12 imbalance alter mRNA expression of neurotrophic genes (BDNF, NGF, TrkB, and CREB)? Would omega-3 fatty acid supplementation be able to modify these effects?	Normal or excess folic acid, with sufficient or deficient vitamin B12. Vitamin B12-deficient groups received omega-3 fatty acid supplementation (DHA + EPA). Half of the dams were switched to a controlled diet after delivery.	Pregnancy	An imbalance in maternal micronutrients reduced mRNA expression of BDNF, NGF, TrkB, and CREB within the adult offspring cortex. Although postnatal diet correction has limited effects, prenatal omega-3 fatty acid supplementation improved/normalized the expression of those genes.
Mahajan [33] PMID: 31772242	Did altered maternal dietary ratios of folate and vitamin B12 change the expression of folate and B12 transporters, microRNAs, and DNA methylation within maternal and fetal tissues?	For 4 weeks before mating, female mice were fed a total of nine different combination of folate and vitamin B12. Offspring continued with the same diets.	Both pregnancy and lactation	The main findings in this study were that altered maternal dietary ratios of folate and vitamin B12 ultimately changed the expression of folate and B12 transporters, as well as dysregulated miR-483/miR-221/miR-133, while also modifying DNA methylation patterns in both maternal and fetal tissues. The deficiencies found within folate and B12 were connected to the increased transporter and DNMT expression that altered methylation patterns, which indicated transgenerational epigenetic effects of micronutrient imbalance.
Ly [21] PMID: 27152363	Which gestational period is likely to be more sensitive to maternal folic acid supplementation, and how would the supplementation affect the tissue folate concentrations, DNA methylation, and gene expression within the rat offspring?	Pregnant rats either received a control diet (2 mg/kg folic acid) or a folic acid supplementation at 2.5 times the control (5 mg/kg) within the gestational weeks.	Pregnancy	The main findings within this study concerned how maternal folic acid supplementation altered tissue folate levels in the offspring. Supplementation during late gestation or throughout pregnancy decreased DNA methylation within the brain and ultimately reduced Er-α, Gr, and Ppar- α gene expression in the liver.
Barua [17] PMID: 24484737	This study investigated whether high maternal folic acid throughout pregnancy alters DNA methylation and gene expression within the brains of mouse offspring.	C57BL/6J female mice were fed a custom diet. The diet contained either low folic acid or high folic acid, beginning one week prior to mating and continuing throughout gestation.	Pregnancy	This study found that high maternal folic acid throughout gestation caused widespread changes in DNA methylation and also altered gene expression within the offspring brain, while including sex-specific effects.
Xu [34] PMID: 39599606	During the periconceptional period, does high maternal folic acid supplementation alter cell-type-specific transcriptomics, spatial gene expression patterns, and chromatin accessibility within the offspring’s brain?	In this study, female mice were fed a control acid diet (2 mg/kg) or a high folic acid diet (20 mg/kg) starting 2 weeks before mating and continuing throughout pregnancy and lactation.	Both pregnancy and lactation	It was found that high maternal folic acid supplementation caused region- and cell-type-specific changes in gene expression and chromatin accessibility in male offspring at P21. The most changes were found in excitatory neurons of the dentate gyrus, in pathways related to ribosomal function and neurodevelopment.
Dong [35] PMID: 39547266	To determine the role of prenatal choline as a modulator of metabolic health and DNA methylation in liver tissue of offspring.	From day two of gestation, pregnant females were given 0.25% choline (w/w) as choline bitrate until the birth of their offspring.	Pregnancy	It was found that at post-weaning hepatic gene expression of insulin signaling substrates, Irs2 was higher in females, and this continued to 12 weeks, whereas Irs1 was lower in male offspring only at the 12-week timepoint. When DNA methylation was measured at the 12-week timepoint, Irs2 was lower in choline-supplemented females. Male offspring with choline supplementation had higher Irs1 methylation.

## Data Availability

No new data were created or analyzed in this study. Data sharing is not applicable to this article.

## Abbreviations

The following abbreviations are used in this manuscript:

BDNF	Brain-derived neurotrophic factor
DOHaD	Developmental Origins of Health and Disease
DNMT1	DNA methyltransferase 1
DRD4	Dopamine D4 receptor
ER-α	Estrogen receptor-α
GR	Glucocorticoid receptor
IGF2	Insulin-like growth factor-2
LEP	Leptin producing gene
MTHFR	Methylenetetrahydrofolate reductase
NGF	Nerve growth factor
NR3C1	Nuclear receptor subfamily 3 group c member 1
1C	One-carbon
PPAR-α	Peroxisome proliferator-activated receptor-α
RBC	Red blood cell
RXRA	Retinoid x receptor alpha
SAH	S-adenosylhomocysteine
SAM	S-adenosylmethionine
5-HTT	Serotonin transporter
